# Poly-amino acids coated gold nanorod and doxorubicin for synergistic photodynamic therapy and chemotherapy in ovarian cancer cells

**DOI:** 10.1042/BSR20192521

**Published:** 2019-12-23

**Authors:** JinYing Liu, Wei Ma, Wei Kou, Lina Shang, Rui Huang, Jin Zhao

**Affiliations:** School of Medicine, Northwest University for Nationalities, Lanzhou 730030, China

**Keywords:** apoptosis, gold nanorod, layer-by-layer, Ovary cancers, synergistic anticancer effect

## Abstract

In this work, we have successfully designed and formulated a doxorubicin-loaded polypeptide-based multilayer assembled gold nanorod (DH-GNR). We have hypothesized that near-infrared (NIR) laser irradiation of DH-GNR will combine the benefits of chemotherapy and photothermal therapy. The GNR was surface functionalized with poly-glutamic acid (PGA) and poly-l-Lysine (PLL) with a final layer of hyaluronic acid (HA) that could also serve as a targeting ligand toward the overexpressed CD44 receptors in ovarian cancer cells. The zigzag ζ potential of nanoparticle is a proof of successful assembly of alternative polymers on the GNR surface. NIR irradiation exhibited a burst release of drug in pH 7.4 and pH 5.0 buffer conditions. The combination of doxorubicin (DOX)-based chemotherapy and GNR-based photothermal therapy exhibited a synergistic effect in killing the SKOV3 cancer cells. DH-GNR(+NIR) induced a 82.5% apoptosis (combined early and late apoptosis) compared with only 35.2 and 38.5% for DOX or DH-GNR(−NIR) treated cell group. Results clearly suggest that the excessive reactive oxygen species (ROS) generation in DH-GNR (+NIR) might be responsible for the cell apoptosis and cell death. The promising anticancer effect of DH-GNR will be of great potential in the treatment of ovarian cancers and worth further development for treating other malignant tumors.

## Introduction

Ovarian cancer is a leading cause of gynecologic cancer-related deaths and second most cause of cancer-related deaths after breast cancer in women [1,2]. Almost 75% of patients are diagnosed at a very late stage due to lack of initial screening, inadequate awareness, and asymptomatic nature of ovary cancers [3]. Main treatment options include chemotherapy, cytoreductive surgery, and occasional radiation therapy. Although surgical resection is very effective, however it is limited to the accessible tumors and complete removal is not possible in most of the cases [4,5]. The residual tumors pose a severe danger in the relapse of cancer. Systemic administration of chemotherapeutic drugs though effective in complete cancer cure after surgery, however, 90% of patients develop chemotherapy resistance and eventually fall for the cancer death [6]. Besides, chemotherapeutic results in severe organ-related toxicity due to the non-specific distribution results in below-par therapeutic concentrations in the tumor tissues. Doxorubicin (DOX), an anthracycline moiety is one such drug indicated in the treatment of ovarian and many other cancers, however, DOX itself has toxicity concerns such as cardiotoxicity, nephrotoxicity, and myelosuppression [7,8]. Therefore, it is important to design strategy to improve the anticancer effect of DOX and at the same time to decrease its side effects.

One of the effective strategies would be to encapsulate the DOX in a nanocarrier system where it can improve the pharmacological actions of the encapsulated drug while reducing its accumulation in the normal organs [9]. Second strategy would be to use a second component that could contribute and synergize the anticancer effect in ovarian cancers. In this regard, plasmonic gold nanomaterials such as gold nanoparticles, gold nanorod (GNR), gold nanocage, gold nanoshell, and gold nanosphere have great potential in effective photothermal therapy [10,11]. Among all the gold nanomaterials, GNR is very attractive owing to its greater photothermal conversion efficiency and special geometry [12]. The rod-shaped morphology allows the oscillation of conducting electrons on the surface of nanoparticles. At specific wavelength, oscillation of electrons happens in both long and short axes resulting in the creation of longitudinal localized surface plasmon bands (LPSR) [13]. The relaxation process will lead to the conversion of light energy into heat energy in the presence of near-infrared (NIR) laser irradiation. Compared with other gold nanoparticles, GNR has best suitable NIR absorption process and it exhibited efficient NIR photothermal conversion into heat energy required for the photothermal treatment [14]. GNR also minimized photobleaching to surrounding tissues thereby reducing the side effects to the normal tissues [15]. The converted heat energy could potentially increase the local temperature of more than 20°C and thereby induce hyperthermia-induced tumor ablation (∼42°C) or aid the complementary therapies such as chemotherapy or radiation therapy [16]. The large surface area of GNR makes them attractive carrier for surface modifications for co-delivering several types of small molecules or nucleic acids [17,18]. In the present study, we have hypothesized to combine the benefits of GNR and DOX.

To achieve this purpose, layer-by-layer (LbL) assembly was proposed as an ideal strategy to load the chemotherapeutic drug along with DOX. The small molecule is loaded in the alternative polymer layers surrounding the GNR which remain stable in the physiological fluids in the body while external stimuli such as NIR activate the GNR into hyperthermia and simultaneously allow the release of encapsulated DOX in the LbL assembly [19–21]. The combination of plasmonic property of GNR and DOX-based chemotherapy is expected to provide synergistic anticancer effect in the treatment of ovarian cancers. Herein, GNR was prepared by seed-mediated reduction method. The particles were alternatively coated with polyaminoacids (poly-glutamic acid (PGA) and poly-l-Lysine (PLL)) using negative and positive charges, respectively. Hyaluronic acid (HA) was coated as a final layer and serve as a targeting agent toward the CD44-receptor tumor tissues.

The DOX/GNR-based LbL assembly was characterized in terms of physicochemical characterization and biological parameters. The synergistic effect of DOX and GNR was demonstrated by cell viability assay, apoptosis assay, and reactive oxygen species (ROS) analysis.

## Materials and methods

### Materials

Hexadecyltrimethylammonium bromide (CTAB), ascorbic acid, triethylamine (TEA), silver nitrate (AgNO_3_), and hydrogen tetrachloroaurate (III) tetrahydrate (HAuCl_4_.4H_2_O), and trisodium citrate were obtained from Sigma–Aldrich, China. All other organic solvents were of reagent grade and used without further modifications.

### Synthesis of GNR

GNR was synthesized by seed-mediated growth method as reported elsewhere [22]. Briefly, seed solution was prepared by mixing 15 ml CTAB solution (0.1 M) into 0.5 ml HAuCl_4_ solution (0.01 M) followed by the addition of 120 ml ice-cold NaBH_4_ (0.1 M) and kept aside for 2 h. Separately, 45 ml CTAB solution (0.1 M) was added to 1.8 ml of HAuCl_4_ solution (0.01 M) and 0.27 ml AgNO_3_ solution (0.01 M) was added. To this mixture, 0.3 ml ascorbic acid (0.1 M) was added as a reducing agent. To this solution, 1.5 ml of seed solution was added and then reacted for 3 h in a dark atmosphere at 37°C till it turned to reddish pink solution with the growth of GNR. The resulting CTAB-stabilized GNR was collected by centrifugation and re-dispersed in water. The formation of GNR was confirmed by UV-Vis spectrophotometer.

### Preparation of LbL polymer assembly and DOX loading

The polymers (PGA and PLL) were prepared as 1 mg/ml solution in 12 mM NaCl. To 1 ml GNR solution, 0.25 ml PGA solution was added and incubated for 1 h at room temperature. The nanoparticle was centrifuged at 10000 rpm for 15 min and re-suspended in 1 ml of ultrapure water. Next 1 mM of DOX solution (prepared in acetic acid buffer, pH 4.1) was added and incubated for 1 h. The cationic DOX was electrostatically interacted with the negatively surface-charged PGA. The DOX/PGA-GNR nanoparticle was centrifuged at 10000 rpm for 15 min and re-suspended in 1 ml ultrapure water. Followed by 100 µl PLL that was added to the DOX/PGA-GNR nanoparticles and incubated for 1 h respectively and then PLL/DOX/PGA-GNR was centrifuged at 10000 rpm for 15 min and re-suspended in 1 ml ultrapure water. A second layer of PGA was followed as mentioned above followed by DOX incubation. Similarly, a second PLL layer was coated on top of the previous polymer using the LbL technique. Finally, HA was prepared at a concentration of 0.5 mg/ml and 100 µl of HA solution was added and stirred at 500 rpm for 2 h. The final HA/PLL/DOX/PGA/PLL/DOX/PGA-GNR was obtained after the centrifugation process and stored at 37°C chamber.

### Physicochemical characterization of D-GNR

Dynamic light scattering (DLS) method was used to determine the particle size distribution, ζ potential, and polydispersity index (PDI) using Malvern Zetasizer, U.K. The samples were diluted appropriately, and the parameters were measured at 25°C in a triplicate manner. The particle morphology was measured using transmission electron microscope (TEM; JEM- 1200EX II; JEOL, Tokyo, Japan). The particles were counterstained with 2% phosphotungstic acid (PTA) and observed under 100 kV. The DOX loading in the LbL nanoparticle was measured by fluorescence spectrophotometer. Five milligrams of freeze-dried NP was dissolved in 0.5 ml of DMSO and incubated at 37°C for 4 h. The drug concentration was measured at an optical absorption of 485 nm. Drug loading efficiency (LE) and encapsulation efficiency (EE) were calculated using;

LE = Amount of DOX in NP/Weight of NP × 100%
EE = Amount of DOX in NP/Amount of initial DOX added × 100%

### *In vitro* release of DOX

The *in vitro* release of DOX from DH-GNR was studied in different conditions of pH 7.4 and pH 5.0 along with the presence and absence of NIR irradiation. To obtain the release study, 5 mg of DH-GNR (freeze-dried) was packed in dialysis tubes (MWCO 3500) and immersed in 30 ml respective buffer and placed in a rotating shaker at 500 rpm. At designated time interval, 1 ml release medium was withdrawn and replaced with equal amount of new buffer. The two samples were irradiated with NIR laser (808 nm, 1.5 W/cm^2^) for 5 min to observe the influence of NIR irradiation. The amount of DOX released at each time point was measured by UV-Vis spectrophotometer after standard calibration of known concentration of DOX.

### Cell culture and in vitro cytotoxicity assay

SKOV3 cells were purchased from ATCC, MD, U.S.A. and cultured in RPMI-164O medium supplemented with 10% FBS and 1% antibiotic mixture. The cytotoxicity assay was performed by MTT assay. Briefly, SKOV3 cells were seeded in 96-well plate at a seeding density of 1 × 10^4^ cells/well/100 µl and allowed to grow for 24 h. After 24 h, cells were treated with fresh 100 µl of media containing free DOX and DH-GNR at difference concentration equivalent of DOX. After 6-h incubation, DH-GNR group was exposed with NIR laser (808 nm, 1.5 W/cm^2^) for 5 min and continued to incubate for 24 h. The old cell culture medium was discarded and washed two times with fresh PBS. Next, cells were treated with 10 µl of MTT solution containing a stock solution of 5 mg/ml and incubated for 4 h. Later, 100 µl of DMSO was added to each plate to extract the formazan crystal and the absorbance was measured at 570 nm using a microplate reader.

### Cell apoptosis assay

The SKOV3 cells were seeded in 12-well plate at a seeding density of 2 × 10^5^ cells/well/1000 µl and allowed to grow for 24 h. After 24 h, cells were treated with fresh 1000 µl of media containing free DOX and DH-GNR at a fixed concentration of 1 µg/ml and incubated for 6 h. After 6-h incubation, DH-GNR group was exposed with NIR laser (808 nm, 1.5 W/cm^2^) for 5 min and continued to incubate for complete 24 h. The apoptosis of cancer cells were evaluated by Annexin-V/PI staining analysis. The cells were extracted and pelleted and stained with 2.5 µl each of Annexin-V and PI dye and incubated for 15 min. The volume was made up to 1000 µl analyzed for 10000 events using flow cytometer (FACSCalibur, BD Bioscience, San Jose, CA, U.S.A.).

### Hoechst 33342 assay

The SKOV3 cells were seeded in 12-well plate at a seeding density of 2 × 10^5^ cells/well/1000 µl and allowed to grow for 24 h. After 24 h, cells were treated with fresh 1000 µl of media containing free DOX and DH-GNR at a fixed concentration of 1 µg/ml and incubated for 6 h. After 6-h incubation, DH-GNR group was exposed with NIR laser (808 nm, 1.5 W/cm^2^) for 5 min and continued to incubate for complete 24 h. The apoptosis of cancer cells was evaluated by Hoechst 33342 staining analysis. The cells were stained with Hoechst 33342 (10 µg/ml) for 10 min and incubated in dark conditions at 37°C. The cells were washed thrice with PBS and fixed with 4% paraformaldehyde (PFA) and washed again two times with PBS. The cell images were observed under fluorescence microscope (Nikon A1, Japan).

### Intracellular ROS assay

The intracellular ROS assay was performed by DCFH-DA assay protocol. The SKOV3 cells were seeded in 12-well plate at a seeding density of 2 × 10^5^ cells/well/1000 µl and allowed to grow for 24 h. After 24 h, cells were treated with fresh 1000 µl of media containing free DOX and DH-GNR at a fixed concentration of 1 µg/ml and incubated for 6 h. After 6-h incubation, DH-GNR group was exposed to NIR laser (808 nm, 1.5 W/cm^2^) for 5 min and continued to incubate for complete 24 h. The cells were extracted, washed with PBS, and then incubated with DCFH-DA at a concentration of 8 µg/ml for 30 min at standard incubator. The fluorescence intensity of DCFH-DA which is proportional to the level of ROS is evaluated by flow cytometer. The fluorescence histogram were quantified and presented in the ‘Results’ section.

### Statistical analysis

Statistical analysis of data was performed with the one-way ANOVA (SPSS software, version 13.0, SPSS Inc) to calculate *P*-values. The results were expressed as mean ± SD (SD represents standard deviation), and *P*<0.05 was considered statistically significant.

## Results and discussion

### Preparation and characterization of LbL-based DH-GNR

**Figure 1:**
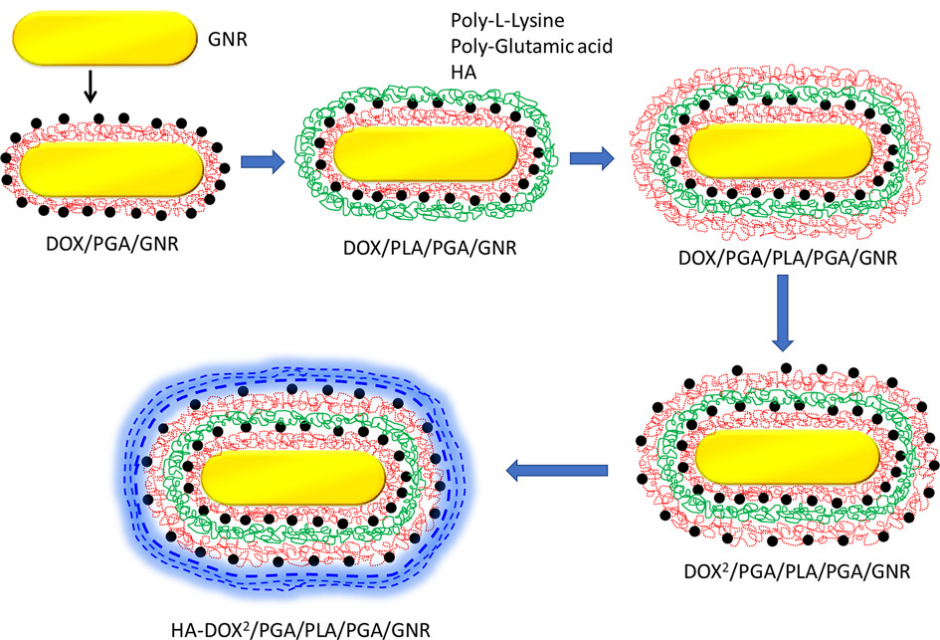
Schematic representation of LbL coating of poly-amino acids on GNR surface HA was coated as a final targeting ligand layer.

**Figure 2:**
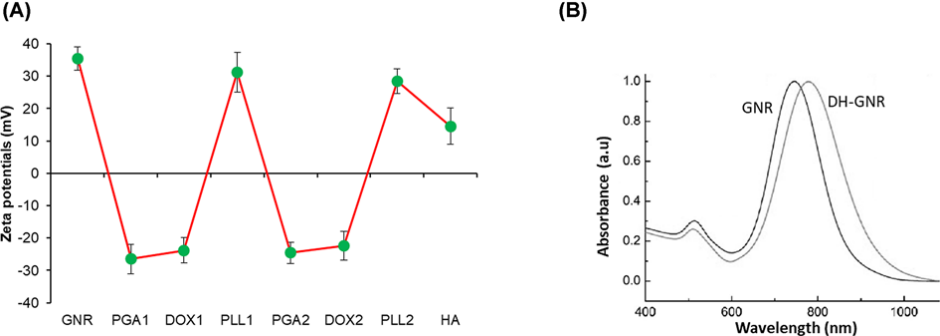
Physicochemical characterizations of nanoparticles (**A**) ζ potential surface charge characterization of bare GNR and LbL coating of each polymer layer. ζ potential was characterized by DLS. (**B**) UV-Vis characterization of GNR and DH-GNR.

In the present study, combination of DOX and GNR was employed as a novel treatment strategy for the ovarian cancers. GNR was prepared by seed-mediated method and the average size of resulting GNR was 40 nm × 5 nm. Furthermore, GNR was wrapped up in a multiple polymer layer with a purpose of minimizing the toxicity associated with the outer CTAB layer as well as to improve the colloidal stability of metallic nanoparticles like GNR. For this purpose, GNR was LbL coated with anionic PGA and PLL and finally coated with HA as targeted layer (Figure 1). The DOX was added and loaded after every anionic layer wherein positively charged DOX will complex with the negatively charged PGA. The LbL assembly of PGA and PLL on GNR surface was monitored by surface charge characterization. As shown, CTAB-stabilized CTAB exhibited a strong positive charge that allowed the deposition of first PGA layer owing to the interaction of positive and negative charges. After first layer PGA deposition, net surface charge of PGA/GNR was negative. The protonated amine group of DOX was utilized to adsorb on the negatively surface charged PGA layer, however, it did not result in charge reversal owing to small charge compensation and low molecular weight of small molecules. To achieve the charge reversal, cationic PLL was deposited as a second layer resulting in a charge reversal of approximately +30 mV indicating the success of polymer deposition. Similarly, third PGA layer and fourth PLL layer was deposited and a characteristic zigzag ζ potential clearly reflect the alternative deposition of polymer materials on the GNR surface indicating the assembly of a definite polymer mass on the metallic nanoparticles (Figure 2A). The positive charge of fourth PLL layer was used to assemble HA as a final polymer layer on the nanoparticles. The presence of outer HA layer will allow the favorable interaction with the CD44 receptor overexpressed cancer cells and might result in the enhanced cellular internalization. The DOX exhibited a high entrapment efficiency of 89.4 ± 1.65% with an active drug loading of 15.8 ± 1.42% w/w indicating the abundance potential of LbL-based nanosystem. Next, UV-Vis spectra of GNR and DH-GNR were observed. It can be clearly noted that the longitudinal plasmon absorption peak of GNR shifted after the LbL deposition of polymers (Figure 2B). The polymer deposition resulted in the changes in the dielectric properties of the GNR thereby confirming the definitive presence of polymers on GNR surface. Different refractive index (RI) of polymers resulted in the shift of the plasmon bands and a progressive damping of longitudinal LPSR peak was clearly observed after five layers of polymer deposition on GNR.

**Figure 3:**
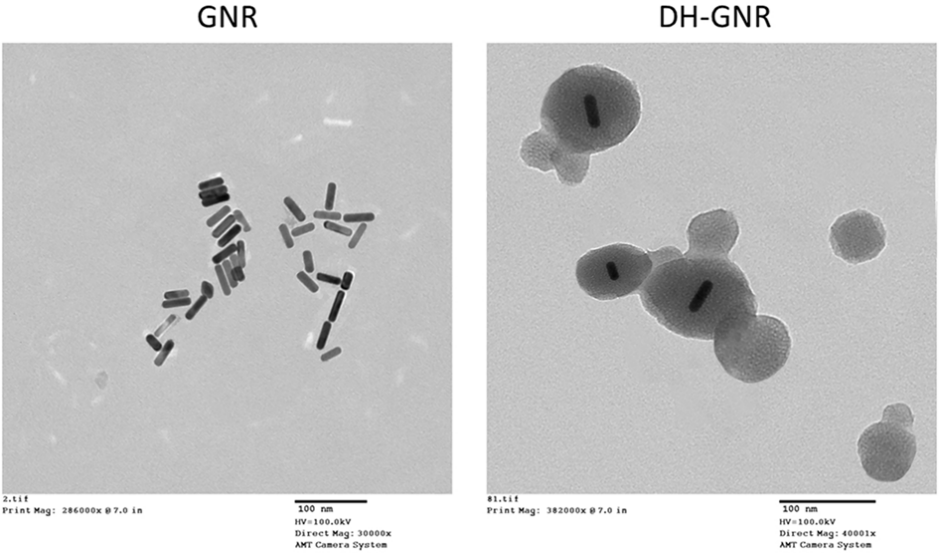
TEM images of GNR and DH-GNR

The morphology of GNR and DH-GNR was evaluated by TEM (Figure 3). As shown, GNR exhibited perfect rod shaped particles with a length of 40 nm and width of 5 nm. The morphology of DH-GNR clearly revealed the LbL assembly of multiple polymer layers. LbL assembly of polymers around the GNR is reflected as a gray shell with a dark rod shaped core with an average colloidal size of 80 nm. Presence of definite polymer shell surrounding the GNR will enhance the stability of metal nanoparticles while it will enhance the prospect of tumor tissue accumulation in the systemic circulations.

### Drug release pattern in the presence and absence of NIR

**Figure 4:**
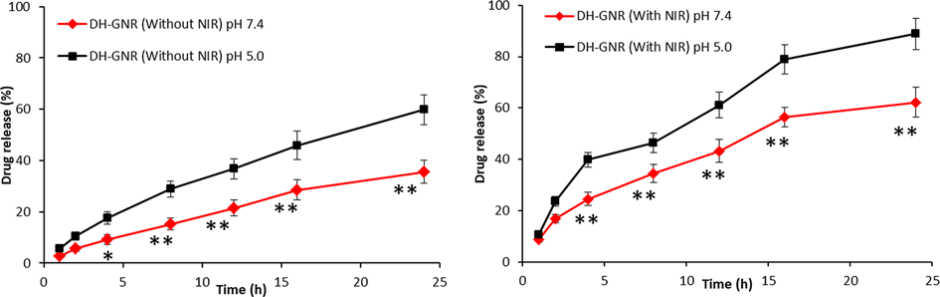
DOX release pattern from DH-GNR at pH 7.4 and pH 5.0 buffer conditions The release of DOX was studied in the presence of NIR irradiation in both pH conditions. *P<0.05 and **P<0.001 is the statistical difference between pH 7.4 and pH 5.0.

The release profile of DOX from DH-GNR was observed in pH 7.4 and pH 5.0 conditions. As shown (Figure 4), a sustained release of drug was observed under pH 7.4 buffer conditions while a relatively faster drug release was observed under acidic conditions. Although no pH-responsive elements are present in the DH-GNR, faster drug release of DOX might be attributed to the basic nature of the drug which provoked a faster drug release in lower pH conditions. As seen, approximately, 35% of DOX released in pH 7.4 conditions after 24 h compared with ∼60% drug release in pH 5.0 conditions. On the contrary, burst release of drug was observed under the influence of NIR irradiation in both the pH conditions. Approximately, 60% of DOX released in 12 h and ∼90% DOX released at the end of 24 h in acidic conditions clearly indicating the influence of NIR irradiation on the DH-GNR. When the NIR irradiation matches the longitudinal LPSR peak of GNR, local temperature will increase leading to the release of drug from the assembled layers. The increase in the local temperature will destabilize the polymer layers leading to the release of encapsulated DOX. Simultaneous action of DOX and NIR-based GNR will exhibit the synergistic anticancer effect in cancer cells.

### *In vitro* cytotoxicity assay

**Figure 5:**
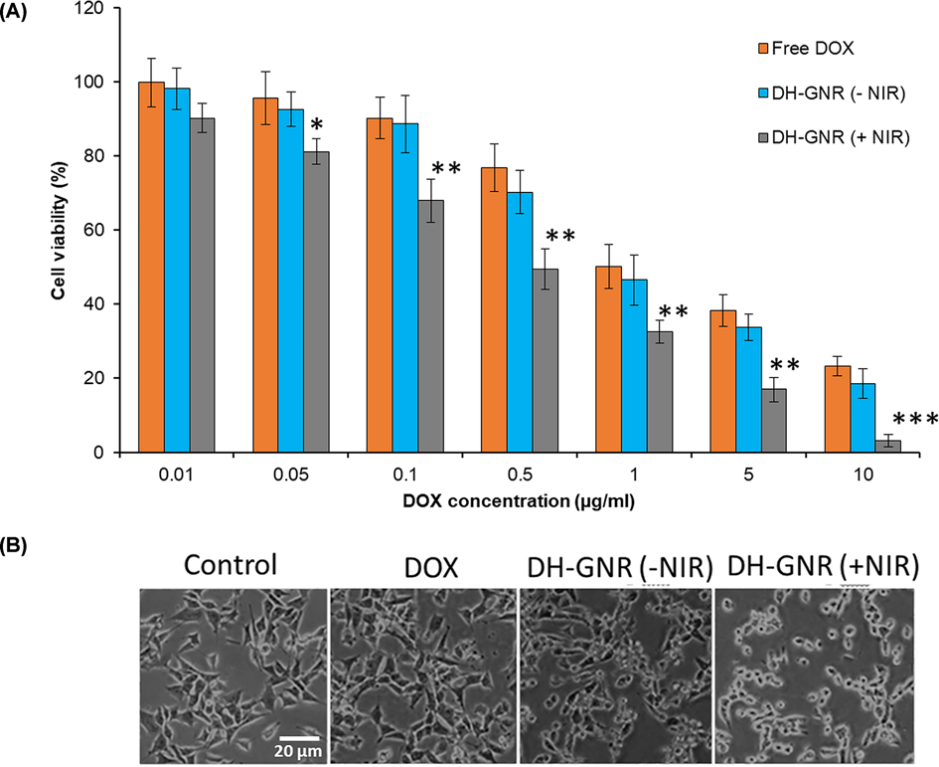
Cytotoxic potential of DH-GNR (**A**) Cellular viability of SKOV3 cancer cells in the presence of DOX, DH-GNR(−NIR), and DH-GNR(+NIR). The cells were incubated for 24 h and cell viability was evaluated by MTT assay protocol. (**B**) Morphological characterization of cell morphology after exposure to above formulations. *P<0.05, **P<0.01 and ***P<0.001 between DH-GNR (-NIR) and (+NIR).

The *in vitro* cytotoxicity assay was evaluated in SKOV3 ovarian cancer cells. The cancer cells were treated with free DOX and DH-GNR (with and without NIR) and incubated for 24 h. As shown, free DOX resulted in a typical concentration-dependent cytotoxic effect in the cancer cells as reported elsewhere (Figure 5A). Notably, DH-GNR(−NIR) showed insignificant difference in cell viability to that of free DOX further reiterating the fact the GNR are non-functional or non-cytotoxic and hold good biocompatibility in the absence of NIR exposure. However, in the presence of NIR irradiation of 1.5 W/cm^2^, significant decrease in the cell viability was observed from DH-GNR compared with the absence of NIR irradiation. For example, cell viability remained at 46.5% for DH-GNR(−NIR) compared with 32.5% for DH-GNR(+NIR) at a base concentration of 1 µg/ml DOX in SKOV3 cancer cells. The IC_50_ value was observed at 1.35, 1.31, and 0.35 µg/ml for free DOX, DH-GNR(−NIR), and DH-GNR(+NIR), respectively. The cytotoxic effect of formulations was further observed by cell morphology analysis (Figure 5B). The greater decrease in cell viability in DH-GNR(+NIR) was possibly attributed to the combinational action of hyperthermia-mediated DOX release and hyperthermia-mediated GNR action at 808 nm laser irradiation. Results imply that combination of photothermal therapy and chemotherapy could be proposed as a treatment strategy in ovarian cancer treatment.

### Apoptosis analysis

**Figure 6:**
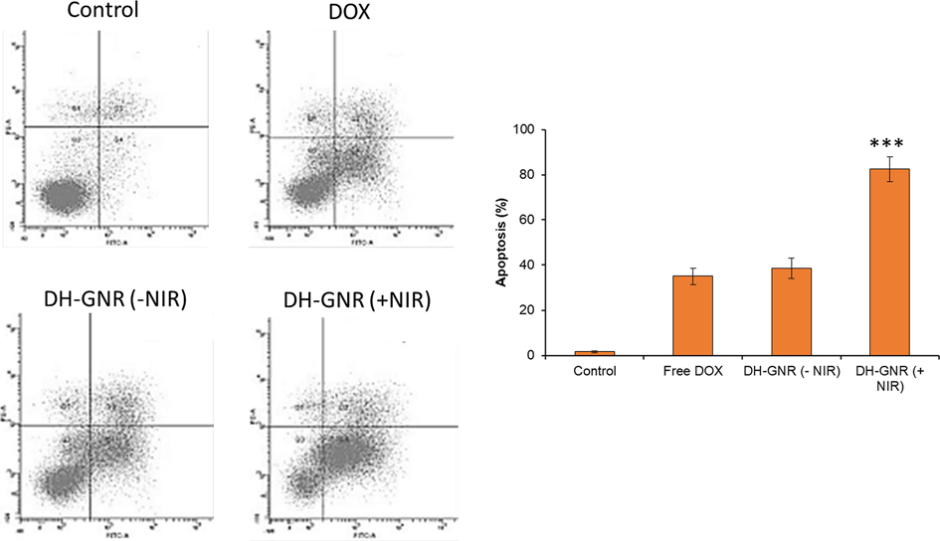
Apoptosis analysis of SKOV3 cells after staining with Annexin-V and PI using flow cytometer The apoptosis has been characterized by early apoptosis, late apoptosis, and necrosis. ***P<0.001 is the difference between -NIR and +NIR.

**Figure 7:**
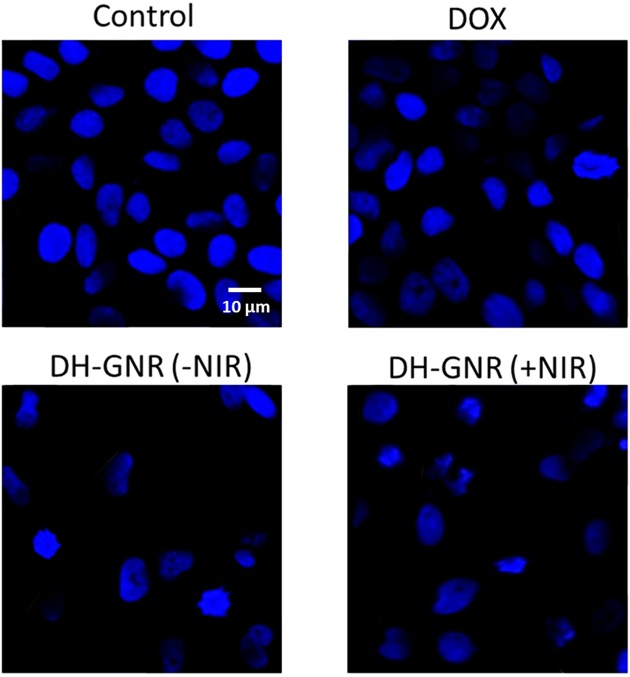
Apoptosis analysis of SKOV3 cancer cells after staining with Hoechst 33342 dye Nuclear morphology was observed under fluorescence microscope.

Cell apoptosis studies were further carried out to confirm the potent anticancer effect of DOX + GNR. As shown, a synergistic photothermal+chemotherapy effect was observed in DH-GNR(+NIR) group (Figure 6). The proportion of apoptotic cells were two-fold higher in DH-GNR(+NIR) treated cell group compared with that of DOX or DH-GNR(−NIR) treated cell group confirming the superior anticancer effect of the combinational regimen. GNR was not effective in killing cancer cells or inducing cell apoptosis in the absence of NIR irradiation. DH-GNR(+NIR) induced a 82.5% apoptosis (combined early and late apoptosis) compared with only 35.2 and 38.5% for DOX or DH-GNR(−NIR) treated cell group. The NIR irradiation to GNR converted the optical energy into thermal energy and induced an enhanced apoptosis of cancer cells. Flow cytometer results clearly indicated that chemotherapy and photothermal therapy synergistically induced cell apoptosis consistent with the cell viability findings. The apoptosis potential was further confirmed by Hoechst 33342 staining analysis. The control cells were intact with normal morphology and DOX did not result in appreciable damage to the cancer cells. DH-GNR(+NIR) however showed a much condensed, fragmented, and distorted cell nuclei (Figure 7). The remarkable apoptosis body formation in DH-GNR(+NIR) treated cancer cell might be attributed to the photodynamic activity rising from the combination of DOX+GNR. It has been reported that apoptosis may be the preferred mechanism of cell death induced by GNR-mediated PDT. The results indicate that the high cytotoxicity induced by DH-GNR might be strongly mediated by apoptosis induction.

### Flow cytometer-based ROS analysis

**Figure 8:**
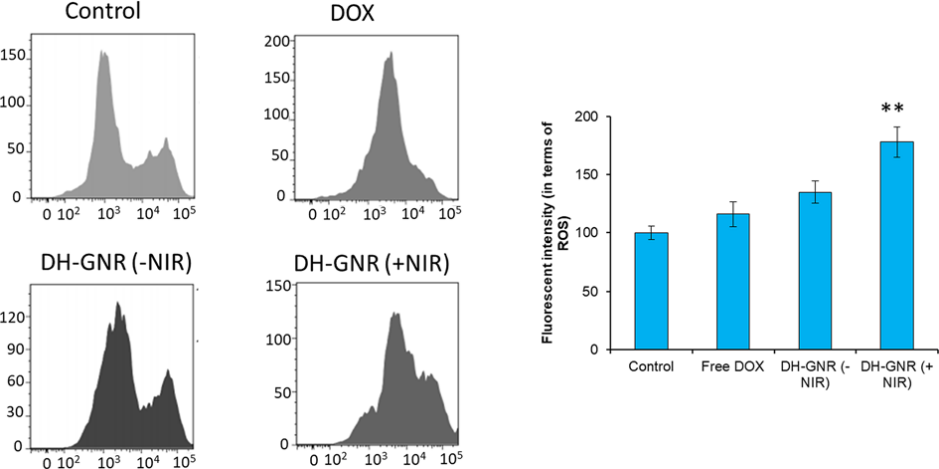
ROS analysis of SKOV3 cancer cells after treatment with DOX, DH-GNR(−NIR), and DH-GNR(+NIR) The ROS analysis was performed by DCFH-DA assay protocol using flow cytometer. **P<0.01 is the statistical difference between -NIR and +NIR.

Next, we investigated the mechanism of cause of apoptosis induction of DH-GNR. As shown, free DOX resulted in negligible ROS induction and almost similar level of ROS was observed in DH-GNR(−NIR) group indicating no photoactivation (Figure 8). In contrast, DH-GNR(+NIR) induced a two-fold higher ROS production in the cancer cells as seen by the drift in the flow cytometer histogram. The excessive generation of ROS is consistent with the decrease in the cell viability of SKOV3 ovarian cancer cells. Results clearly suggest that the excessive ROS generation in DH-GNR(+NIR) might be responsible for the cell apoptosis and cell death. It has been reported that GNR-based photodynamic activity results in increasing the Bax/Bcl-2 expression and GNR acts on mitochondria and allow the release of cytochrome *c* (cyt *c*) into the cytoplasm that will in turn activate the range caspase cascade. The higher release of cyt *c* in the cytosol activates the caspase-3 and caspase-9 and activates the cleavage of PARP which leads to the apoptosis of cancer cells [23,24].

## Conclusion

In this work, we have successfully designed and formulated a DH-GNR. The zigzag ζ potential of nanoparticle is a proof of successful assembly of alternative polymers on the GNR surface. The combination of DOX-based chemotherapy and GNR-based photothermal therapy exhibited a synergistic effect in killing the SKOV3 cancer cells. The IC_50_ value was observed at 1.35, 1.31, and 0.35 µg/ml for free DOX, DH-GNR(−NIR), and DH-GNR(+NIR), respectively. DH-GNR(+NIR) induced 82.5% apoptosis (combined early and late apoptosis) compared with only 35.2 and 38.5% for DOX or DH-GNR(−NIR) treated cell group. Results clearly suggest that the excessive ROS generation in DH-GNR(+NIR) might be responsible for the cell apoptosis and cell death. The promising anticancer effect of DH-GNR will be of great potential in the treatment of ovarian cancers and worth the further development for treating other malignant tumors. Antitumor efficacy analysis and biodistribution characterization on clinically relevant animal model will be a part of next study.

## Abbreviations

CTAB: hexadecyltrimethylammonium bromide
cyt *c*: cytochrome *c*
DH-GNR: doxorubicin-loaded HA-coated gold nanorod
DOX: doxorubicin
EE: encapsulation efficiency
GNR: gold nanorod
HA: hyaluronic acid
LbL: layer-by-layer
LE: loading efficiency
LPSR: localized surface plasmon band
NIR: near-infrared
PGA: poly-glutamic acid
PLL: poly-l-lysine
ROS: reactive oxygen species
TEM: transmission electron microscope
